# Multifunctional Zn(II) Coordination Polymer as Highly Selective Fluorescent Sensor and Adsorbent for Dyes

**DOI:** 10.3390/ijms24108512

**Published:** 2023-05-10

**Authors:** Mohd. Muddassir, Abdullah Alarifi, Naaser A. Y. Abduh, Waseem Sharaf Saeed, Abdulnasser Mahmoud Karami, Mohd. Afzal

**Affiliations:** Department of Chemistry, College of Science, King Saud University, Riyadh 11451, Saudi Arabia

**Keywords:** Schiff base, X-ray crystallography, acetone, fluorescence sensing, adsorptions

## Abstract

A new Zn(II)-based coordination polymer (**1**) comprising the Schiff base ligand obtained by the condensation of 5-aminosalicylic acid and salicylaldehyde has been synthesized. This newly synthesized compound has been characterized by analytical and spectroscopic methods, and finally, by single-crystal X-ray diffraction technique in this study. The X-ray analysis reveals a distorted tetrahedral environment around the central Zn(II) center. This compound has been used as a sensitive and selective fluorescent sensor for acetone and Ag^+^ cations. The photoluminescence measurements indicate that in the presence of acetone, the emission intensity of **1** displays quenching at room temperature. However, other organic solvents caused meagre changes in the emission intensity of **1**. Additionally, the fluorescence intensity of **1** has been examined in the presence of different ketones viz. cyclohexanone, 4-heptanone, and 5-nonanone, to assess the interaction between the C=O group of the ketones and the molecular framework of **1**. Moreover, **1** displays a selective recognition of Ag^+^ in the aqueous medium by an enhancement in its fluorescence intensity, representing its high sensitivity for the detection of Ag^+^ ions in a water sample. Additionally, **1** displays the selective adsorption of cationic dyes (methylene blue and rhodamine B). Hence, **1** showcases its potential as an excellent luminescent probe to detect acetone, other ketones, and Ag^+^ with an exceptional selectivity, and displaying a selective adsorption of cationic dye molecules.

## 1. Introduction

The Schiff bases (SBs), which are regarded as the condensation products of aldehydes (or ketones) and primary amines, results in the production of azomethine or imine groups (−C=N−), that can undergo coordination to the metal ion through its azomethine nitrogen [1]. The ease of the syntheses of the Schiff bases makes them suitable candidates for a wide range of applications in biology [2,3], in organic syntheses, catalysis [4], magnetochemistry [5,6], and superconductivity [3]. Among all the recognized organic sensors, Schiff bases are an assorted class of chemosensor that have been broadly used in the sensing of toxic metal ions (Cd^2+^, Pb^2+^, Hg^2+^ etc.), different beneficial metals (Al^3+^, Zn^2+^, Cu^2+^, Ag^+^, etc.), and organic solvents (acetone, methanol, ethanol, etc.) as industrial waste materials, which pose threats to human health and the environmental ecosystems [7,8].

Amongst the plethora of organic solvents, acetone as a solvent is harmful to both human beings and our environment that could be accredited to its easy volatilization, toxicity, and flammable nature [9]. It is present in the environment in small quantities, but a significant amount of it (97%) is released into the atmosphere during its production, which can quickly enter the water and the soil via rain and snow. Its exposure leads to kidney, liver, and nerve damage, increased congenital disabilities, and a lowered ability to reproduce (for males only) [10].

Silver is relatively more abundant than other precious metal ions; therefore, it is less expensive than other valuable metals, such as gold. As such, it is widely used in industry and daily life, as well as in medicine. For example, when silver salts are supplemented into antibiotics, their efficiency increases dramatically [11]. Silver can also decrease bacterial resistance, enabling the antibiotic to kill them quickly [12]. However, an excess amount of Ag^+^ ion may cause allergic reactions in the body, which has been recognized as a contraindication for using silver in medical devices or antibiotics. Furthermore, colloidal silver may also co-operate with some medications, thereby reducing the absorption of certain antibiotics and thyroxine [13]. Silver-based materials are regularly consumed in the process of manufacturing silver door handles, solar panels, coins, electronics, optics, photographic equipment, bio-sensing devices, jewelry, etc. This increases the risk of the absorption of Ag+ ions, resulting in their unnecessary deposition into tissues.

Moreover, thousands of tons of silver present in various forms are released into the environment annually, which is toxic to flora and fauna. This is due to the fact that while low concentrations of silver can be used for the disinfection and preservation of drinking water, at high levels (>0.9 μM), silver can trigger toxic effects [14,15,16]. The excess intake of silver has been observed to be risky to human health, causing brain damage and immune system disruption, as they easily undergo complexation with amino acids and nucleic acids, showing a high affinity toward sulfhydryl enzymes, and affecting ATP functions in fish and zooplankton [17,18,19].

Thus, it is of paramount importance to design efficient methods that can detect acetone and Ag^+^ ions at sub-ppm levels to overcome their environmental and human health concerns. In the past few decades, several detection tools have been employed for the detection of solvents, which include various analytical techniques, Raman spectroscopy, and electrochemical methods. However, these methods are not only expensive and time-consuming, but also require complicated procedures to be carried out. Among them, fluorescence techniques have gained notable attention in the detection of environmentally hazardous materials owing to their high sensitivity, quick response, timely detection, cost-effectiveness, and simple process. Amongst the variety of fluorescent materials, the metal complexes derived from the Schiff bases are apt candidates to be used as sensors for the sensitive and selective detection of the different classes of analytes.

Dyes are highly stable organic compounds that have found a wide industrial application in paper, leather, silk, cotton, and food, and are involved in our daily life. However, the degradation of the dyes in wastewater discharge is not easy, and hence, its treatment is of paramount importance. Therefore, it is very important to develop an efficient and reliable method to treat the wastewater discharge containing dyes to protect the aqueous environment as well as human and aquatic life health.

In this context, transition metal complexes obtained from Schiff bases that are capable of forming supramolecular interactions can act as appropriate fluorescence sensors against harmful solvents including acetone, ethanol, methanol, nitro explosives, and metals [20,21,22,23,24,25]. Sun et al. described a d^10^-based metal system that displayed a sensitive and selective sensing of acetone and displayed multifunctional characteristics [26]. Previous studies have revealed that metal complexes having coordinated with lattice solvent molecules may hinder the solvents/nitro explosives/metals from interacting with metal ions [27]. Hence, the exclusion of such solvent molecules in the coordination sphere and its lattice is necessary for the development of effective fluorescence sensors for practical purposes as the presence of such solvent molecules in the lattice systems require further pre-activation.

In view of these noteworthy facts associated with the Zn(II)-Schiff base complexes and the associated benefits of the d^10^-configuration [28] based Zn(II) complex, these complexes can act as sensors for a varied type of analytes viz. acetone and analogous solvents/nitro explosives/different metals. In the present investigation, we have successfully synthesized a new Zn(II)-based Schiff base complex (**1**) using the condensation products of 5-aminosalicylic acid and salicylaldehyde, characterized it, and used it as a fluorescent sensor against acetone, as a turn-on sensor for Ag^+^ cations, and as an apt adsorbent for cationic dyes viz. methylene blue and rhodamine B. The pertinent results of these investigations are presented henceforth.

## 2. Results and Discussion

**Scheme 1:**
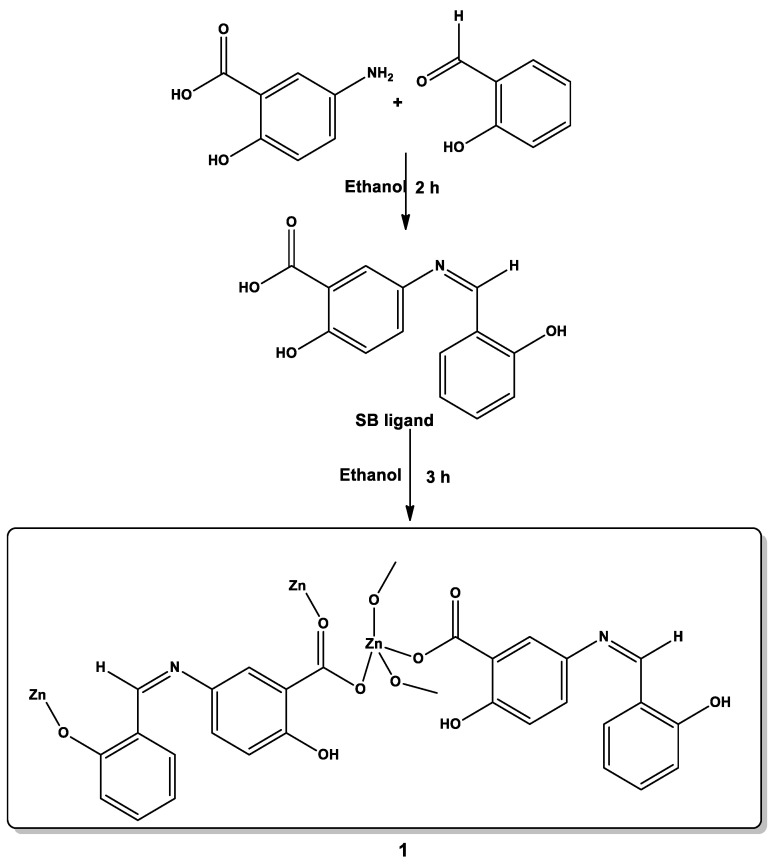
Synthetic routes for the preparation of complex **1**.

The SB ligand was synthesized by condensing 5-aminosalicylic acid with salicylaldehyde in ethanol, which on reacting with zinc acetate, yielded the desired Zn(II)-based coordination polymer **1** (Scheme 1). The polymer was characterized by microanalyses, FTIR, UV–Vis, ^1^H and ^13^C NMR spectroscopy, and thermogravimetric analysis, and was air and moisture stable. The coordination polymer was soluble in acetonitrile and other organic solvents, but insoluble in petroleum ether and diethyl ether.

### 2.1. Structural Description of ***1***

**Figure 1:**
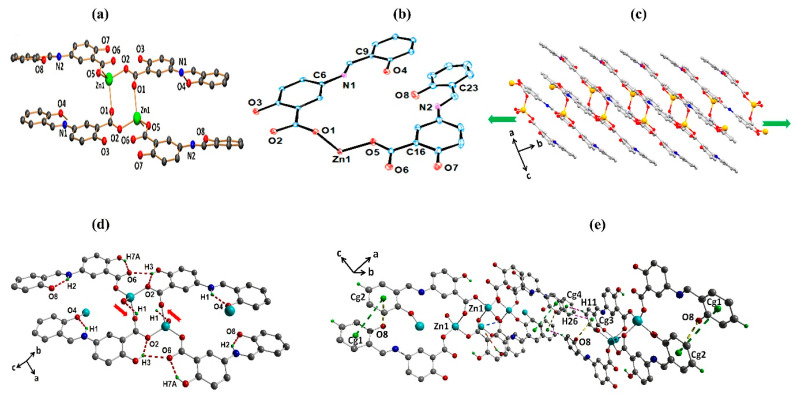
(**a**) The perspective view of **1**. (**b**) ORTEP diagram for the asymmetric unit of **1** with a 30% probability density. (**c**) Two-dimensional layered packing of **1**. (**d**) Strong N–H⋯O and O–H⋯O hydrogen bonding interactions in **1**. (**e**) Varied non-covalent interactions in **1** (color catalogs: π⋯π green, C–O⋯π yellow, C–H⋯π pink, and Zn⋯O blue).

The crystals suitable for single crystal X-ray diffraction were obtained by the slow evaporation of acetonitrile solution. The single crystal X-ray analysis revealed that complex **1** crystallizes in the monoclinic crystal system with the *P*2_1_/*n* space group. The asymmetric unit in **1** comprises two SB ligands mutually bridged by Zn(II) (Figure 1a). The immediate geometry around the Zn(II) is distorted tetrahedrally, which is satisfied by the carboxylate–oxygen centers of the SB ligand. Each Zn(II) is coordinated with four carboxylate–oxygen centers of four SB ligands with an average Zn–O bond distance of ~1.95(2) Å, and an average bond angle O–Zn–O′ of 106.57(8)°. These geometrical parameters are consistent with the previously reported Zn(II) complexes [22,29,30]. The two Zn(II) centers constitute an eight-membered crown structure by coordinating with the oxygen atoms of the carboxylate group of the SB ligand (Figure 1a). In this ring, the separation between the two Zn(II) centers is 4.159 Å [31]. The eight-membered puckered crown also encompasses several internal bond angles which were as follows: O1–Zn1–O2 = 101.76°, O1–C1–O2 = 123.05°, Zn1–O2–C1 = 121.68°, and Zn1–O1–C1 = 126.40°, which together, indicates significant angle strain.

Furthermore, it was found that due to the layer-by-layer stacking of the molecules, a two-dimensional incrusted framework developed along the *b*-axis in **1** (Figure 1c). The extra oxygen atoms that were not involved in coordination with the Zn(II) center exhibited inter-layer coordination with the next vacant coordination site of the Zn(II) atom to engender a 2D-layered coordination polymeric framework. Moreover, the solid-state structure of **1** was stabilized by varied non-covalent interactions. The flexible aromatic rings of the Schiff base ligands were involved in π⋯π stacking interactions, with a separation of 3.900 Å counterbalancing the overall angle strain in the eight-membered crown structure. Also, both mild and robust hydrogen bonds further imparted stability upon the crown.

Crystal structure analyzes revealed that the oxygen atoms of both the phenolic and carboxylic groups of **1** were found to be involved in the intramolecular hydrogen bonding N1–H1⋯O4 interaction, with a distance of 1.875 Å and a N1–H1⋯O4 angle of 137.25°. Similarly, another N2–H2⋯O8 was found with a 1.685(3) Å distance, with a N2–H2⋯O8 angle of 137.83°. Apart from N–H⋯O interactions, O7–H7A⋯O6 also exists in **1**, with an interaction distance of 1.813 Å and having an O7–H7A⋯O6 angle of 145.56°. Additionally, two oxygen’s, O2 and O6, also underwent interactions with the bond H3 center. The presence of two lone pairs of electrons on O6, and the ease of accessibility of the donor hydrogen atom, provoke such unusual bonding interactions, with an O3–H3⋯O2 distance of 1.901 Å and an O3–H3–O2 angle of 142.61°; and O3–H3⋯O6 with distance of 2.353(3) Å and an <O3–H3–O6 angle of 130.93° (Figure 1d).

Molecules were further interlocked by other interactions viz. C–H⋯π and C–O⋯π, that transformed the 2D framework into three-dimensional supramolecular assemblies by interlocking the 2D layers along the *ab* plane. The O8 center was involved in the C–O⋯π interaction, with the Cg2 centroid of an aromatic ring having had an interaction distance of 3.642 Å, and an angle C8–O8–Cg2 of 85.63° (Figure 1e). The Zn(II) center ion was also involved in the Zn⋯O interaction, with a distance of 3.054 Å and bond angle Zn–O–Zn of 108.9°.

**Figure 2:**
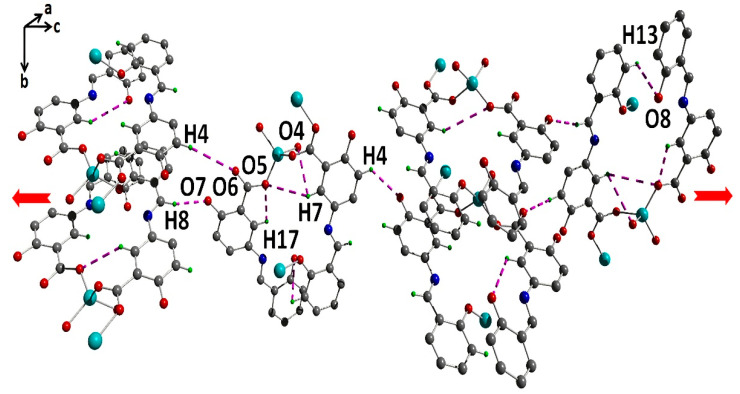
Representing weak C–H⋯O hydrogen bonding interactions in complex **1**.

Apart from this, other weak intramolecular C–H⋯O hydrogen bonding interactions were also present in **1** that contributed to the formation of the integrated network arrangement of molecules (Figure 2). This was due to the presence of two lone pairs on the oxygen atom, and the easy accessibility of nearby hydrogen atoms which promoted the formation of a bifurcated hydrogen bonding from donor O5 to acceptors H7 and H17, with a H7⋯O5 and H17⋯O5 bond distances of 3.392 Å and 2.391 Å, respectively with C7–H7–O5 and C17–H17–O5 bond angles of 141.51° and 100.92°, respectively. The molecules were further locked by C–H⋯O interactions such as H13⋯O8, with a distance of 3.561 Å, and a C13–H13–O8 angle of 72.74°. Likewise, the H7⋯O4 interaction was found to be 3.536 Å long and has a C7–H7–O4 interaction angle of 86.76°. Also, H8⋯O7 was found to exist, which was 2.296 Å long with a C8–H8–O7 angle of 164.76° (Figure 2). All these weak non-covalent interactions, due to the interlocking of the molecules in a specific orientation, played a dominant role in fixing the supramolecular assembly in the preferred direction of the axis.

### 2.2. Hirshfeld Surface Analysis

**Figure 3:**
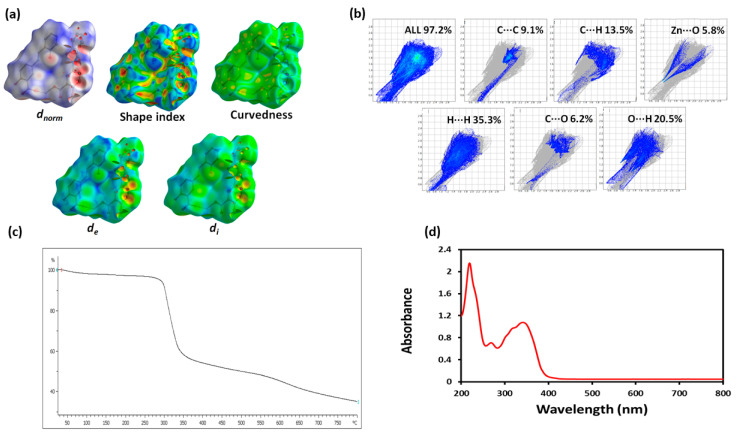
(**a**) Hirshfeld surfaces of **1**; (**b**) Fingerprint plots of **1**; (**c**) TGA of **1**; (**d**) UV–Vis. spectrum for **1**.

Hirshfeld surface analysis is a powerful tool used to explain the intramolecular interactions within the crystal by offering 3D viz. *d*_norm_, shape index, curvedness, and 2D images such as fingerprint plots [32,33]. The Hirshfeld surfaces for **1** is presented in Figure 3a, which show surfaces mapped with the *d*_norm_ range −1.894 Å to 1.272 Å, the shape index range from −1 Å to 1 Å, and the curvedness range from −4 Å to 0.4 Å, respectively. The 2D fingerprint plots permit the rational interpretation of the nature of the intermolecular interactions present in **1** (Figure 3b), and also provide quantitative insights regarding the non-covalent interactions.

The *d*_norm_ surface of **1** possesses pale blue patches, which indicate that most of the supramolecular interactions involved in building the architecture were isoenergetic in nature. However, while the red regions on the *d*_norm_ surface indicate the dominance of N–H⋯O, O–H⋯O, and C–H⋯O hydrogen bonding interactions, the light blue areas indicate the weaker non-covalent interactions. The curvedness plot, which was derived from the function of the root mean square of surface curvature, displayed green regions with light blue patches, which is characteristic of C–H⋯π and π⋯π stacking, while the flat surface with slight globular depressions indicates hydrogen bonding interactions.

The fingerprint plots for **1** revealed sharp spikes or teeth, which are the signature of the N–H⋯O and O–H⋯O hydrogen bonding interactions. The H⋯O interactions contribute 20.5% of the total Hirshfeld surface, while the C⋯H and C⋯C interactions contributes 13.5% and 9.1%, respectively, of the total Hirshfeld surface area. Also, the interesting Zn⋯O and C⋯O interactions were found to have 5.8% and 6.2% contributions, respectively, of the Hirshfeld surface area of **1**. Apart from these interactions existing in the crystal structure of **1**, the H⋯H interactions also contributes dominantly, with a net contribution of 35.3% of the total Hirshfeld surface.

### 2.3. FTIR Spectroscopy

In the FTIR spectrum of **1** (ESI, Figure S1), the stretching vibrations characteristic of azomethine υ(C=N) appears at 1609 cm^−1^. The unaltered position of this band as observed for the uncoordinated ligand, suggested no coordination of azomethine nitrogen to Zn(II). Also, bands at 1655 and 1426 cm^−1^ were typically assigned to the antisymmetric (ν_as_) and symmetric (ν_s_) vibrations corresponding to the COO^−^ carboxylate groups, respectively [30]. In general, the Δν (ν_as_(CO_2_)–ν_s_(CO_2_)) value is used to analyze the coordination mode of carboxylate to the metal center [34]. In **1**, the Δν value is greater than 200 cm^−1^, thereby indicating a monodentate coordination mode of the carboxylate moiety with Zn(II). The medium-to-weak intensity bands at 3042 and 3430 cm^−1^ corresponds to the –CH and OH stretching vibrations, respectively, while a sharp intense band at 1493 cm^−1^ could be assigned to the aromatic –CH bending modes [35]. Also, υ(Zn–O) in the FTIR spectrum of **1** appeared as a medium intensity band at 441 cm^−1^ [36].

### 2.4. Thermal Studies

Furthermore, to assess the thermal stability, thermogravimetric analysis (TGA) for **1** was performed between 25–800 °C under a N_2_ atmosphere (Figure 3c). The TGA plot indicated that in **1**, weight loss occurred in three steps. In the first step, the loss of volatile components occurred in the temperature range 50–275 °C, while in the second step, loss of the organic components of the SB ligand took place between 280–340 °C. The third and final weight loss eventually led to the formation of zinc oxide as the final product.

### 2.5. Electronic Absorption Spectroscopy

In the electronic absorption spectrum of **1**, the high-energy bands at 221, 270, and 342 nm rose due to the intraligand and metal-to-ligand charge transfer (MLCT) transitions, respectively (Figure 3d). Also, because of the presence of the d^10^-configuration in Zn(II), no low energy *d-d* transitions appeared in **1** [37].

### 2.6. NMR Spectroscopy

The purity and composition of **1** was assessed with the aid of ^1^H NMR spectroscopy (Figure S2). In the ^1^H NMR spectrum of **1**, the resonance, due to free carboxylic (–COOH) groups at 12.0–10.0, were absent, thereby indicating the deprotonation of the –COOH group, and their concomitant coordination with the Zn(II) center. Meanwhile, the signal formed as a result of the phenolic (–OH) group appeared at 13.08 ppm. Also, resonances at 8.97 ppm and 7.81–6.95 ppm arose due to the CH=N and aromatic protons, respectively. In the ^13^C NMR spectrum of **1**, resonances appearing at 171.1, 156.3, and 147.5 ppm can be attributed to the C=O, CO, and C=N moieties. Additionally, resonances corresponding to the aromatic carbon centers of the SB ligand arose at between 118.3–135.3 ppm. The appearance of additional ^13^C resonances can be ascribed due to the C–C coupling in the SB ligand.

### 2.7. Mass Spectrometry

In the mass spectrum of **1**, a low-intensity molecular ion peak appeared at *m*/*z* 316, corresponding to C_14_H_8_NO_4_Zn^−^, while the base peak was observed at *m*/*z* 256 for C_14_H_10_NO_4_^−^. Hence, the base peak was found to be related to the Schiff base (SB) fragment (Figure S3).

### 2.8. Fluorescence Sensing Property

The coordination of the d^10^-configuration metal ions with the conjugated ligands has no influence on the emission profile of such ligands. On the contrary, other metal ions exhibit quenching effects in different solvents because of their different electronic configurations [38]. To assess this previously reported interpretation, in this study, the influence of solvents on the emission behavior of **1** was investigated.

**Figure 4:**
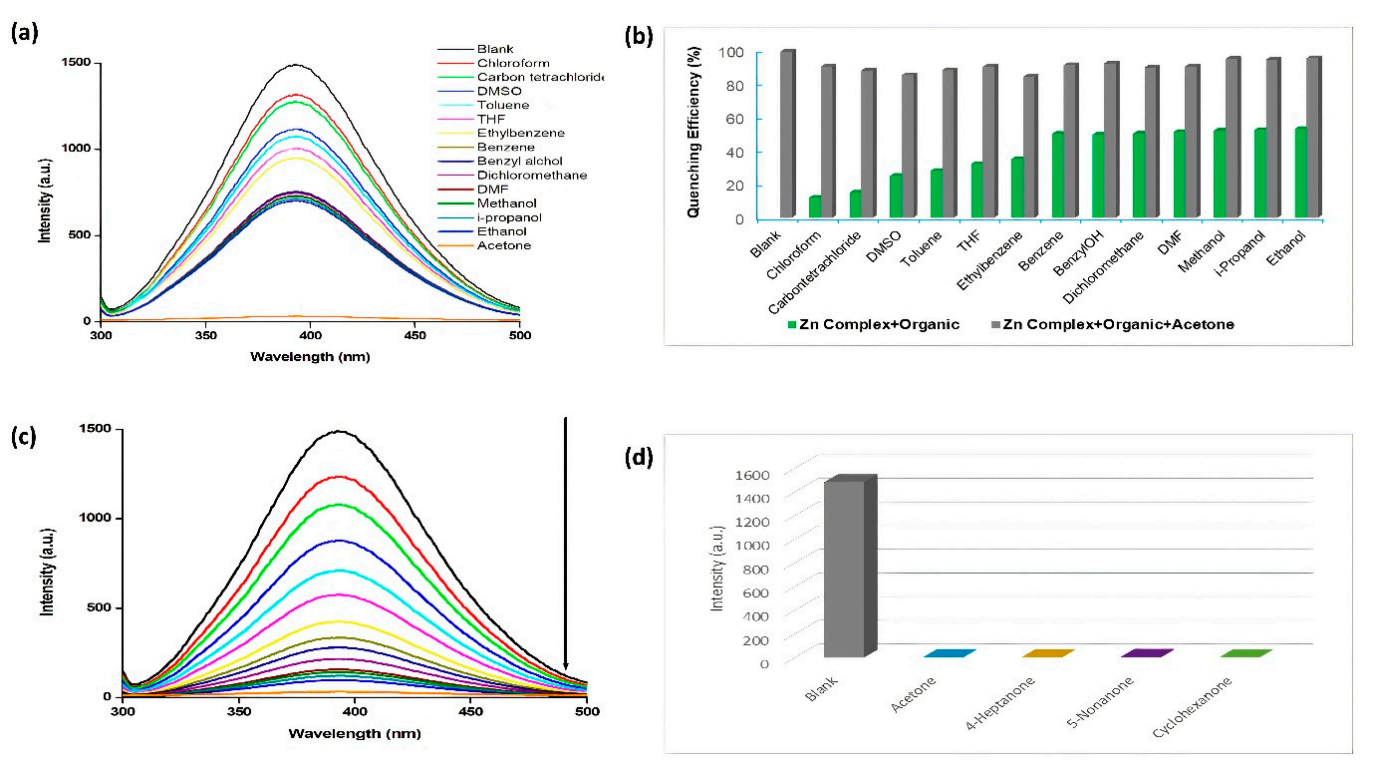
(**a**) Fluorescence spectra of complex **1** in acetonitrile (blank) and in the presence of various other solvents. (**b**) Quenching efficiency of complex **1** upon the addition of various organic solvents, except for acetone (green) and the subsequent addition of acetone (grey). (**c**) Change in the fluorescence intensity of **1** dissolved in acetonitrile (blank) upon titration with acetone. (**d**) Fluorescence intensity of complex **1** in various ketones (λ_ex_ = 270 nm).

The emission study in acetonitrile solution revealed that on excitation at 270 nm, **1** exhibited intense emission at 393 nm (Figure 4a). Also, previous reports indicated that the rational designing of the ligand can increase the rigidity of the aromatic backbone of the ligands, and in turn, can enhance the intra/intermolecular interactions, thereby favoring the energy transfer [39]. Hence, the emissive behavior of **1** was assessed in a series of organic solvents (methanol, ethanol, isopropanol, benzyl alcohol, chloroform, dichloromethane, carbon tetrachloride, benzene, toluene, THF, ethylbenzene, DMF, DMSO, and acetone).

Figure 4a shows that amongst all the organic solvents, the addition of acetone quenched the fluorescence emission of **1** to the maximum extent (98%). Moreover, the addition of the alcohols led to the decline in the fluorescence intensity up to ~50% compared to acetone. Hence, it can be concluded that **1** can be used as a turn-off sensor against acetone.

In an actual system, the selective detection of a particular solvent from a mixture of solvents is essential to assess the suitability of the material as sensor against a specific solvent molecule. Hence, interference experiments were performed to check the selectivity of **1** as a sensor against acetone. The interference experiments revealed no perceptible changes in the performance of **1** as a turn-off sensor against acetone in the presence of other solvents (Figure 4b). Therefore, **1** could be employed as a selective and sensitive sensor for the detection of acetone. Importantly, **1** does not require pre-activation, such as heating or pH maintenance, to be employed as a sensor against acetone in contrast to lanthanides [40], and other coordination polymers [27].

Furthermore, thorough analysis to check the sensing ability of **1** against acetone was executed with varying concentrations of acetone (Figure 4c). The results indicated a decline in the fluorescence emission intensity of **1** on the addition of increasing amounts of acetone. Based on these experiments, the Stern–Volmer plot was constructed (Figure S4a), which exhibited a linear relationship at low concentration values with the Stern-Volmer constant (K_sp_) 1.94 × 10^4^ M^−1^ and *R*^2^ = 0.9964, which is better than previously reported fluorescent sensors for acetone [26,28]. The limit of detection for acetone was calculated from the 3σ/slope [41] (σ: standard deviation), that was of the order of 5.5 × 10^−6^ mol L^−1^ (*R*^2^ = 0.9886) (ESI, Figure S4b), and is lower than previously reported fluorescence sensors for acetone [20,26,28].

In order to explore the quenching in the emission of **1** with acetone, the fluorescence behavior of **1** in the presence of other ketones (C=O), such as cyclohexanone, 4-heptanone, and 5-nonanone, was examined (Figure 4d). The results revealed that the quenching in the emission intensity of **1** may arise due to the oxophilic interactions operating between the C=O group of ketones and the frameworks of **1**. Also, the decline in the emission intensity may have occurred due to the competitive absorption of the light energy between the ketones and the Schiff base ligand framework of **1**. Hence, it can be concluded that quenching operates via the transfer of energy from the SB ligand of **1** to the acetone molecules [42,43].

### 2.9. Detection of Metal Ions

As the azomethine (CH=N) nitrogen remains uncoordinated to the Zn(II) ions in **1**, the suitability of **1** as a sensor for metal ions was also investigated. This is because, on addition of different metal ions, they can coordinate azomethine nitrogen, and in turn can alter the spectral response of **1**.

**Figure 5:**
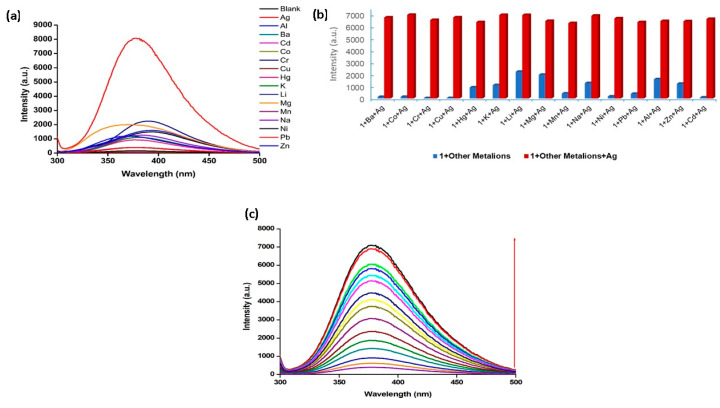
(**a**) Fluorescence spectra of complex **1** in acetonitrile (blank) and in the presence of different metal ions (λ_ex_ = 270 nm); (**b**) in the presence of various metal ions (red) and without Ag^+^ ions (sky blue); (**c**) upon incremental addition of Ag^+^ ions (5 × 10^−6^ to 6.0 × 10^−5^ M).

To probe the ability of **1** in sensing metal ions, aqueous solutions of MCl_x_ (M = Ba^2+^, Co^2+^, Cr^3+^, Cu^2+^, Hg^2+^, K^+^, Li^+^, Mg^2+^, Na^+^, Ni^2+^, Pb^2+^, Al^3+^, Zn^2+^, Cd^2+^, and Ag^+^) were prepared by dissolving nitrate salts in triple-distilled water, and these were titrated against **1** (1.0 × 10^−5^ M), and the emission profiles of these solutions were then recorded. The experiments indicated perceptible enhancements in the fluorescence intensity in the presence of Ag^+^ ions (turn-on), while other metal ions offered almost negligible changes in the emission intensity of **1** (Figure 5a). Also, a decline in fluorescence intensity of **1** occurred in the presence of Cu^2+^ (turn-off), and these findings were deemed to be consistent with those for other previously reported Zn(II)–Schiff base complexes, suggesting on–off–on switching [44]. The intense emissions of **1** in the presence of Ag^+^ ions indicated its use as a chemosensor for distinguishing Ag^+^ ions in a mixture containing other metal ions.

The precise recognition of specific metal ions in a mixture of other ions is crucial. For this purpose, an aqueous solution of Ag^+^ ions was added into **1**, already containing the aqueous solutions of different metal ions, and the changes in the fluorescence intensities were subsequently monitored (Figure 5b). The results indicated that the emission intensity of complex **1** in the presence of Ag^+^ ions remained almost unaltered in the presence of other metal ions. Therefore, the competitive analysis shows that the detection of Ag^+^ by **1** may not be affected by the presence of other metal ions, which further showcases the potential of **1** as a highly selective sensor for Ag^+^ ions.

Moreover, to explain the sensitivity and detection of Ag^+^ ions by **1**, another experiment was carried out by varying the concentration of Ag^+^ ions (5 × 10^−6^ to 6.0 × 10^−5^ M) (Figure 5c). On the basis of these experiments, a fluorescence titration profile was constructed (ESI, Figure S5a), and using the Stern–Volmer equation, a linear relationship between intensity and concentration was observed with a 1:1 binding model and the binding constant of 5.8 × 10^3^ M^−1^ (*R*^2^ = 0.9939). The corresponding limit of detection was found to be 1.5 × 10^−7^ M (*R*^2^ = 0.9964) (as shown in ESI, Figure S5b), which is better than previously reported fluorescent probes for Ag^+^ [19,44,45]. The low detection limit is permitted by the guidelines of the World Health Organization (WHO) for drinking water [46], and by the U.S. EPA [47]. Hence, overall, it can be concluded that **1** is a promising sensor that can be used to distinguish between safe and toxic levels of Ag^+^ ions in drinking water.

Past studies have indicated that silver usually forms complexes with a coordination number two [48]. In this study, Ag^+^ can interact with the oxygen and azomethine nitrogen centers of the SB ligand of **1**, which also favors the 1:1 stoichiometry based on the linear relationship of intensity with concentration in the abovementioned fluorescence titration experiment. Also, there is another possibility of the displacement of Zn(II) by the Ag^+^ ion, but failure in the attempts to isolate the silver complex by the direct reaction of AgNO_3_ with similar Zn(II)–Schiff base complexes indicated that such metal–ion substitutions are not feasible under normal circumstances [49,50,51].

### 2.10. Repeatability and Recyclability

The recyclability of **1** as a sensor for acetone and Ag^+^ ions was also performed. As shown in Figure S6a,b (ESI), no significant alterations in the emission properties of **1** were observed in either case (acetone and Ag^+^ ions) after three cycles of experiments. This suggested that **1** can be utilized repeatedly as a turn-off sensor for acetone, and a turn-on sensor for Ag^+^ ions. Meanwhile, the PXRD pattern of **1** in Ag^+^ was found to match well with the simulated PXRD pattern obtained from the single crystal data. Although there is a slight broadening in the PXRD pattern of Ag^+^@**1**, the major peaks remained unaltered in the experimental PXRD pattern, which indicates the excellent stability and robustness of **1** in an aqueous medium under these conditions (ESI, Figure S7).

### 2.11. Adsorption of Dyes

**Figure 6:**
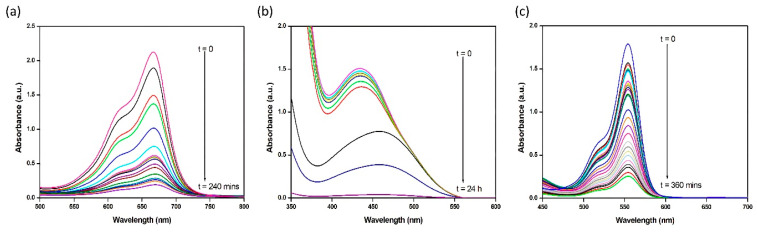
Changes in the UV–Vis absorption pattern of complex **1** in the presence of (**a**) MB, (**b**) MO, and (**c**) Rh-B as a function of time.

A major environmental concern is the discharge/release of various dangerous chemicals by the dye and textile industries, which degrade soils and eventually contaminate drinking water [52]. In addition to degrading the color of the water, dyes also slow down photosynthesis and lower the levels of dissolved oxygen, which undoubtably have an impact on all types of aquatic life [53]. In light of this, several methods have been used to remove dyes from the environment. However, because of its efficacy, economical nature, and productivity, removal using suitable adsorbents such as employing coordination-based polymers is the most suitable process [26]. Hence, the properties of **1** dye adsorbent were assessed using the model dyes viz. methylene blue (MB) and rhodamine B (RhB) as cationic dyes, and methyl orange (MO) as an anionic dye. The dyes MB, RhB, and MO exhibited their UV–Vis. absorption maxima at 665, 550, and 460 nm, respectively. The adsorption experiments indicated that the absorption intensities of these dyes decrease gradually in the presence of adsorbent **1** with exposure time (Figure 6), and that the adsorbent **1** was capable of adsorbing these dyes up to ~95% within the exposure timeframe. In comparison to anionic dyes, which required almost 24 h to be entirely adsorbed by **1**, both cationic dyes were efficiently absorbed by **1** within 15 min and needed less time (4–6 h) to be totally adsorbed by **1**.

Based on these results, it can be concluded that **1** possesses a stronger ability to adsorb cationic dyes than anionic dyes. Since, all the dyes are linear, but the molecular size of MO is smaller compared to the other two cationic dyes, this may explain the lower adsorption of MO by **1**. Moreover, there may be strong π···π interactions between the aromatic backbone of **1** and these dyes, that might result in their efficient adsorption. In addition, careful studying of the crystal structure of **1** suggests that it possesses electron-free –OH and –Oxo sites, which can interact better with cationic dyes than with anionic dyes. This might therefore be the reason for why **1** exhibited a strong adsorption ability towards the cationic dyes.

## 3. Materials and Methods

### 3.1. Starting Materials

5-Aminosalicylic acid (≥99%, Sigma-Aldrich, St. Louis, MO, USA), salicylaldehyde (98%, Sigma-Aldrich) and zinc acetate (99.99% trace metals basis, Sigma-Aldrich) were all used as received. All solvents, including methanol, ethanol, isopropanol, isobutanol, chloroform, dichloromethane, carbon tetrachloride, benzene, toluene, ethylbenzene, acetonitrile, dimethyformamide, ethyl acetate, acetone, cyclohexanone, 4-heptanone, and 5-nonanone (Sigma-Aldrich), were all used as received.

### 3.2. Synthesis

#### 3.2.1. Synthesis of the Schiff Base Ligand (SB)

The SB ligand was prepared in accordance with the previously reported procedure of Bourque et al. [54]. The compounds 5-aminosalicylic acid (0.25 g, 1.63 mmol) and salicylaldehyde (0.22 g, 1.80 mmol) were refluxed in ethanol (30 mL) for two hours to obtain the yellow precipitate. The obtained precipitate was then filtered and washed with ethanol (3 × 2.5 mL) and diethyl ether (3 × 5 mL), and vacuum dried to afford SB.

Yield, 75%, m.p. 258–260 °C. IR (Nujol, cm^−1^) ν: 1610 (C=N).

#### 3.2.2. Synthesis of the Zn(II) Complex (**1**)

Complex **1** was synthesized by refluxing the SB ligand (0.29 g, 1.15 mmol) and Zn(CH_3_COO)_2_ (0.20 g, 1.09 mmol) in ethanol (30 mL) for 3 h. The resulting yellow product was filtered, washed with a 1:1 ether and EtOH solution, and dried in vacuo. Yield, 57% (0.621 mmol), m.p. 263 °C. Anal. Calc. for C_28_H_20_N_2_O_8_Zn (%) C, 58.20; H, 3.49; N, 4.85, Found: C, 58.48; H, 3.41; N, 4.77. ∧_M_ (1 × 10^−3^ M, CH_3_CN): 19.0 Ω^−1^cm^2^ mol^−1^ (non-electrolyte).

^1^H NMR (DMSO-*d*_6_, ppm): 13.08 (br, s, 2H, –OH), 8.97 (s, 2H, C(H)=N), 7.81 (d, *J* = 2.4, 2H Ar), 7.63 (m, *J* = 3.2 Hz 6H, Ar), 7.40 (d of d, *J* = 0.8, 7.2 Hz, 2H, Ar), 7.02 (t, *J* = 4.4 Hz, 4H, Ar). ^13^C NMR (DMSO-d_6_, ppm): 171.1 (C=O), 156.3 (CO), 147.5 (C=N), 135.3, 128.9, 128.7, 127.3, 125.5, 123.6, 123.4, 123.1, 122.9, 121.4, 119.2, 118.9 (ArC).

### 3.3. Sensing and Dye Adsorption

The detailed methodologies for the acetone and cation sensing, as well as dye adsorption, are presented in the supporting information.

### 3.4. X-ray Crystallography

The crystallographic refinement data are presented in Table 1. Selected bond distances and angles are listed in Tables S1 and S2, respectively (CCDC number 1974273).

## 4. Conclusions

In summary, a new Zn(II)-based coordination polymer comprising Schiff base ligands has been synthesized and used as a sensitive and selective fluorescent sensor for acetone and Ag^+^ cations. The photoluminescence measurements indicate that in the presence of acetone, the emission intensity of the polymer displays quenching at room temperature. Additionally, the fluorescence intensity of this polymer has been examined in the presence of different ketones viz. cyclohexanone, 4-heptanone, and 5-nonanone, to assess the interactions between the C=O group of ketones and the molecular framework of the polymer. Also, the polymer displays selective recognition of Ag^+^ in the aqueous medium by enhancements in its fluorescence intensity, representing its high sensitivity for the detection of Ag^+^ ions in a water sample. Additionally, it displayed a selective adsorption of cationic dyes (methylene blue and rhodamine B). Hence, it showcases its potential as an excellent luminescent probe to detect acetone, other ketones, and Ag^+^ with an exceptional selectivity, and displaying a selective adsorption of cationic dye molecules. This work provides a new, economically viable, and less complicated synthetic approach for selectively and sensitively estimating acetone and Ag^+^ ions. This study provides new physical insights into the rational design of coordination complex-based functional materials. Future work in this direction will be crucial to elucidate the specific roles of d^10^-based coordination polymers in sensing. Further work is underway to prepare new advanced multifunctional metal complexes as practical and feasible water-soluble sensors, with applications in the chemosensing of the biologically relevant and environmentally significant analytes.

## Figures and Tables

**Table 1:** Crystal data and structure refinement for complex **1**.

CCDC Number	1974273	
Empirical formula	C_28_H_20_N_2_O_8_Zn	
Formula weight	577.83	
Temperature	150 (2) K	
Wavelength	0.71073 Å	
Crystal system	Monoclinic	
Space group	P 21/n	
Unit cell dimensions	a = 16.386 (5) Å	α = 90°
	b = 6.2462 (10) Å	β = 107.669°
	c = 24.188 (6) Å	γ = 90°
Volume	2358.9 (10) Å^3^	
Z	4	
Density (calculated)	1.627 Mg/m^3^	
Absorption coefficient	1.101 mm^−1^	
F(000)	1184	
Crystal size	0.440 × 0.030 × 0.020 mm	
Theta range for data collection	2.5768 to 28.3260°	
Index ranges	−21 ≤ h ≤ 21, −8 ≤ k ≤ 7, −32 ≤ l ≤ 32	
Reflections collected	9984	
Independent reflections	5830 (R(int) = 0.0513)	
Completeness to theta = 25.242°	99.7%	
Absorption correction	Multi-scan	
Max. and min. transmission	0.978 and 0.643	
Refinement method	Full-matrix least-squares on F^2^	
Data/restraints/parameters	5830/568/491	
Goodness-of-fit on F^2^	1.056	
Final R indices (I > 2sigma(I))	R1 = 0.0513, wR2 = 0.0884	
R indices (all data)	R1 = 0.0864, wR2 = 0.0990	

Largest diff. peak and hole: 0.429 and −0.445 e·Å^−3^.

## Supplementary Materials

The following supporting information can be downloaded at: https://www.mdpi.com/article/10.3390/ijms24108512/s1. References [55,56,57,58,59,60] are cited in the supplementary materials.
