# Supplementary material for: Multifunctional Zn(II) Coordination Polymer as Highly Selective Fluorescent Sensor and Adsorbent for Dyes
Source: Int J Mol Sci. 2023 May 10;24(10):8512. doi: 10.3390/ijms24108512 (PMC10217995; doi:10.3390/ijms24108512)
Supplement: Supplementary file 1 [file ijms-24-08512-s001.zip › ijms-2209532-supplementary.pdf]

Supporting Information

Multifunctional Zn (II) coordination polymer as highly selective fluorescent sensor and adsorbent for dyes

Mohd. Muddassir[1]*, Abdullah Alarifi[1], Naaser A. Y. Abduh[1], Waseem Sharaf Saeed[1], Abdulnasser Mahmoud Karami[1], Mohd. Afzal[1]

[1]Department of Chemistry, College of Science, King Saud University, Riyadh 11451, Saudi Arabia

*Correspondence: muddassir@ksu.edu.sa

Experimental

Methods and Instrumentations

The IR spectra were recorded with a VECTOR 22 spectrometer using KBr pellets in the 200–4000 cm^{-1} region. Elemental analyses of C, H and N were performed on a PerkinElmer 240C elemental analyzer. TGA/DSC was performed on Universal V3.8 B TA SDT Q600 Build 51 thermal analyzer under a nitrogen atmosphere using alumina powder as the reference material. Electronic spectra were recorded on UV–1700 PharmaSpec UV–Vis spectrophotometer (Shimadzu). ^{1}H and ^{13}C NMR spectra of complex **1** were recorded at 25 °C on a JEOL 400 NMR spectrometer. Emission spectra were recorded on a Shimadzu RF–6000 fluorescence spectrophotometer.

X-Ray crystal structure determination

Single crystals of complex 1 were coated with a trace of Fomblin oil and quickly transferred to the goniometer head of a Bruker Quest diffractometer with a fixed chi angle, a sealed tube fine focus X-ray tube, single crystal curved graphite incident beam monochromator, a Photon100 CMOS area detector and an Oxford Cryosystems low temperature device. Examination and data collection were performed with Mo Kα radiation (λ = 0.71073 Å) at 150 K.

Data were collected, reflections were indexed and processed, and the files scaled and corrected for absorption using APEX3 [1]. The space groups were assigned and the structures were solved by direct methods using XPREP within the SHELXTL suite of programs [2,3] and refined by full matrix least squares against F^2 with all reflections using Shelxl2018 [4,5] using the graphical interface Shelxle [6]. H atoms attached to carbon and nitrogen atoms as well as hydroxyl hydrogens were positioned geometrically and constrained to ride on their parent atoms. C-H bond distances were constrained to 0.95 Å for aromatic and alkene C-H moieties. N-H bond

distances were constrained to 0.88 Å for planar (sp^2 hybridized) N-H groups. O-H distances of alcohols were constrained to 0.84 Å. Hydroxyl H atoms were allowed to rotate but not to tip to best fit the experimental electron density. $U_{iso}(H)$ values were set to a multiple of $U_{eq}(C)$ with 1.5 for OH, and 1.2 for C-H and N-H units, respectively.

One of the two ligands is disordered by an approximate 180° rotation around the Schiff base C-N single bond. The disorder extends into the two aromatic rings and attached phenol(ate) oxygen atoms. The two disordered moieties were restrained to have similar geometries. U^{ij} components of ADPs for disordered atoms closer to each other than 2.0 Å were restrained to be similar. Subject to these conditions the occupancy ratio refined to 0.758(4) to 0.242(4).

References

1 Bruker (2016). Apex3 v2016.9-0, Saint V8.34A, SAINT V8.37A, Bruker AXS Inc.: Madison (WI), USA, 2013/2014. (2016).

2 SHELXTL suite of programs, Version 6.14, 2000-2003, Bruker Advanced X-ray Solutions, Bruker AXS Inc., Madison, Wisconsin: USA (Bruker Advanced X-ray Solutions, Bruker AXS Inc. Madison, Wisconsin: USA, Madison, Wisconsin: USA).

3 Sheldrick, G. M. (2008).

4 Sheldrick George M. University of Göttingen, Germany. (2018).

5 Sheldrick, G. M. Crystal structure refinement with SHELXL. *Acta crystallographica. Section C, Structural chemistry* **71**, 3-8, doi:10.1107/S2053229614024218 (2015).

6 Hübschle, C. B., Sheldrick, G. M. & Dittrich, B. ShelXle: A Qt graphical user interface for SHELXL. *J. Appl. Crystallogr.* **44**, 1281-1284, doi:10.1107/S0021889811043202 (2011).

Fluorescence Titrations. The fluorescence spectrum of complex **1** was measured in CH_3CN (1.0×10^{-5} M) at 298 K. All titrations were performed by the gradual addition of acetone/Ag^+ in an incremental fashion. Each titration was repeated several times to get concordant value. No shape change but only intensity decrease was observed in the emission spectra during the titration process. The fluorescence quenching efficiency (%) was calculated with $(1 - I/I_0) \times 100$, where I_0 and I are the fluorescence intensities before and after the addition of acetone, respectively.

Selective Dye Removal.

An aqueous stock solution of methyl orange (MO), methylene blue (MB), or rhodamine B (RhB; 1000 ppm) was prepared by dissolving dyes in deionized water. Aqueous solutions of 10 ppm MO, MB, or RhB were obtained by dilution of the stock solution with water. The MO, MB, or RhB concentrations were determined by using absorbance (at 464, 665, and 554 nm, respectively) of the solutions.

Before adsorption, the adsorbents were desolvated and kept in a desiccator. Then, an exact amount of the adsorbents (10 mg) was put in the aqueous dye solutions (20 mL, 10 ppm). The dye solutions containing the adsorbents were mixed well under magnetic stirring and maintained for a fixed time depending on the required time at 25 ◦C in dark. After adsorption for a predetermined time, the solution was separated from the adsorbents, and the dye concentration was calculated according to the absorbance.

Table S1. Bond lengths [Å] for complex **1**.

Zn1-O1	2.004(2)	C2-C7	1.392(4)
Zn1-O5	1.914(2)	C3-C4	1.401(4)
Zn1-O4_a	1.941(2)	C4-C5	1.372(4)
Zn1-O2_c	1.943(2)	C5-C6	1.398(4)
O1-C1	1.259(3)	C6-C7	1.380(3)
O2-C1	1.272(3)	C8-C9	1.422(4)
O3-C3	1.351(3)	C9-C10	1.412(4)
O4-C14	1.326(3)	C9-C14	1.423(4)
O5-C15	1.273(3)	C10-C11	1.366(4)
O6-C15	1.248(3)	C11-C12	1.395(4)
O7-C21	1.365(12)	C12-C13	1.376(4)
O7B-C21B	1.36(4)	C13-C14	1.410(4)
O8-C28	1.346(6)	C15-C16	1.492(4)
O8B-C28B	1.345(17)	C15-C16B	1.492(4)
C16-C21	1.399(11)		
C16-C17	1.387(10)		
C16B-C17B	1.41(3)		
N1-C8	1.306(3)	C16B-C21B	1.41(3)
N1-C6	1.419(4)	C17-C18	1.384(16)
N2-C22	1.280(6)	C17B-C18B	1.39(5)
N2-C18	1.430(7)	C18-C19	1.403(9)
N2B-C22B	1.274(18)	C18B-C19B	1.39(3)
N2B-C18B	1.42(3)	C19-C20	1.372(10)
C19B-C20B	1.34(3)		
C20-C21	1.400(16)		
C20B-C21B	1.40(5)		
C1-C2	1.481(3)	C22-C23	1.444(7)
C2-C3	1.409(4)	C22B-C23B	1.437(19)
C23-C28	1.397(8)		
C23-C24	1.399(8)		
C23B-C28B	1.38(2)		

C23B-C24B	1.39(2)
C24-C25	1.393(10)
C24B-C25B	1.39(3)
C25-C26	1.379(13)
C25B-C26B	1.38(6)
C26-C27	1.349(18)
C26B-C27B	1.35(3)
C27-C28	1.407(10)
C27B-C28B	1.41(3)

Table S2. Bond angles [°] for complex **1**.

O1-Zn1-O5	113.88(8)	O3-C3-C2	124.4(2)
O1-Zn1-O4_a	100.96(8)	C2-C3-C4	119.1(2)
O1-Zn1-O2_c	101.76(8)	O3-C3-C4	116.5(2)
O4_a-Zn1-O5	112.17(9)	C3-C4-C5	121.3(2)
O2_c-Zn1-O5	111.39(8)	C4-C5-C6	119.6(2)
O2_c-Zn1-O4_a	115.88(8)	C5-C6-C7	119.8(2)
Zn1-O1-C1	126.40(17)	N1-C6-C7	116.8(2)
Zn1_c -O2-C1	121.68(17)	N1-C6-C5	123.4(2)
Zn1_b-O4-C14	124.86(16)	C2-C7-C6	121.4(2)
Zn1-O5-C15	123.97(19)	N1-C8-C9	123.2(2)
C3-O3-H3	109.00	C10-C9-C14	119.7(2)
C21-O7-H7A	109.00	C8-C9-C14	121.8(2)
C21B-O7B-H7B	109.00	C8-C9-C10	118.6(2)
C6-N1-C8	128.4(2)	C9-C10-C11	121.0(3)
C18-N2-C22	124.3(5)	C10-C11-C12	119.5(3)
C18B-N2B-C22B	124.1(15)	C11-C12-C13	121.2(3)
C12-C13-C14	120.8(3)		
O4-C14-C13	122.5(2)		
C9-C14-C13	117.9(2)		

O4-C14-C9	119.6(2)		
O5-C15-C16B	115.8(3)		
O5-C15-C16	115.8(3)		
O1-C1-O2	123.1(2)	O5-C15-O6	124.8(3)
O2-C1-C2	116.1(2)	O6-C15-C16	119.4(3)
O1-C1-C2	120.9(2)	O6-C15-C16B	119.4(3)
C3-C2-C7	118.8(2)	C15-C16-C17	119.1(5)
C1-C2-C3	122.2(2)	C17-C16-C21	120.0(8)
C1-C2-C7	119.0(2)	C15-C16-C21	120.9(6)
C15-C16B-C17B	126.7(15)	C22B-C23B-C28B	121.0(13)
C17B-C16B-C21B	116(2)	C23-C24-C25	120.0(6)
C15-C16B-C21B	117.3(17)	C23B-C24B-C25B	120.2(19)
C16-C17-C18	121.6(7)	C24-C25-C26	119.9(8)
C16B-C17B-C18B	122(2)	C24B-C25B-C26B	119(2)
C17-C18-C19	117.7(6)	C25-C26-C27	121.3(9)
N2-C18-C17	117.0(5)	C25B-C26B-C27B	123(3)
N2-C18-C19	125.0(7)	C26-C27-C28	119.7(7)
C17B-C18B-C19B	119(2)	C26B-C27B-C28B	118(3)
N2B-C18B-C19B	117(2)	O8-C28-C27	117.7(7)
N2B-C18B-C17B	122(2)	C23-C28-C27	120.3(6)
C18-C19-C20	121.6(7)	O8-C28-C23	122.0(5)
C18B-C19B-C20B	119(2)	C23B-C28B-C27B	120.9(14)
C19-C20-C21	120.2(7)	O8B-C28B-C23B	123.0(14)
C19B-C20B-C21B	121.8(19)	O8B-C28B-C27B	116.0(16)
C16-C21-C20	118.8(7)		
O7-C21-C16	120.9(11)		
O7-C21-C20	120.3(8)		
C16B-C21B-C20B	120(2)		
O7B-C21B-C20B	118(3)		
O7B-C21B-C16B	121(3)		
N2-C22-C23	121.2(4)		
N2B-C22B-C23B	121.3(13)		
C24-C23-C28	118.6(5)		
C22-C23-C28	121.7(5)		

C22-C23-C24	119.7(5)
C24B-C23B-C28B	119.3(14)
C22B-C23B-C24B	119.7(13)

Symmetry transformations used to generate equivalent atoms:

1#	x, y, z
2#	1/2-x, 1/2+y, 1/2-z
3#	- x, - y, -z
4#	1/2+x, ½-y, ½+z

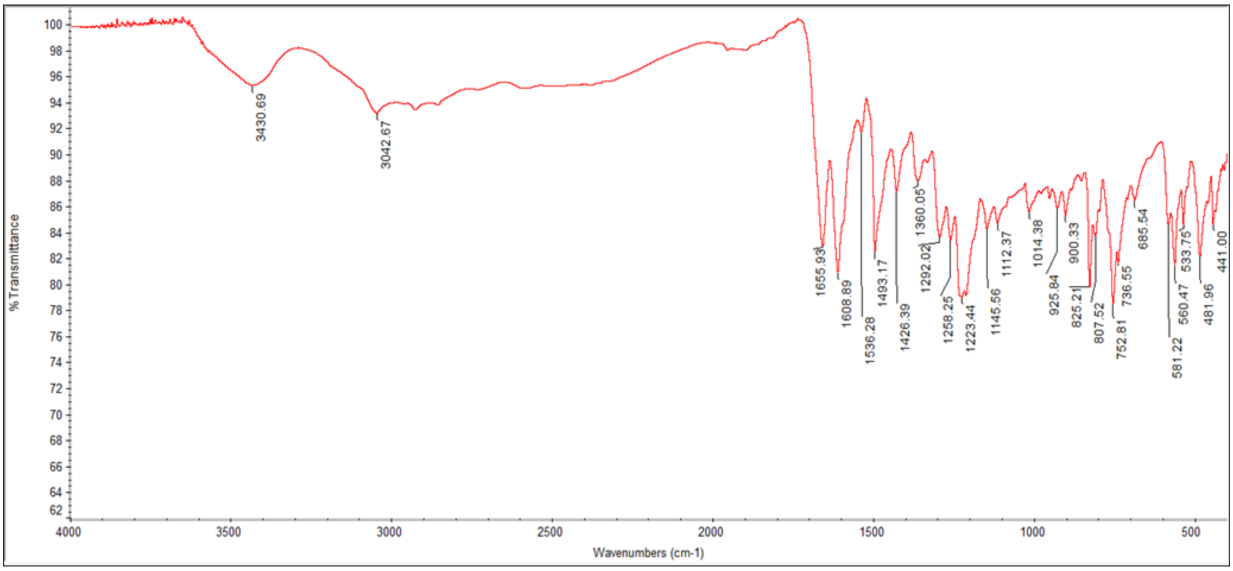

Fig. S1- FT-IR spectrum of complex **1**.

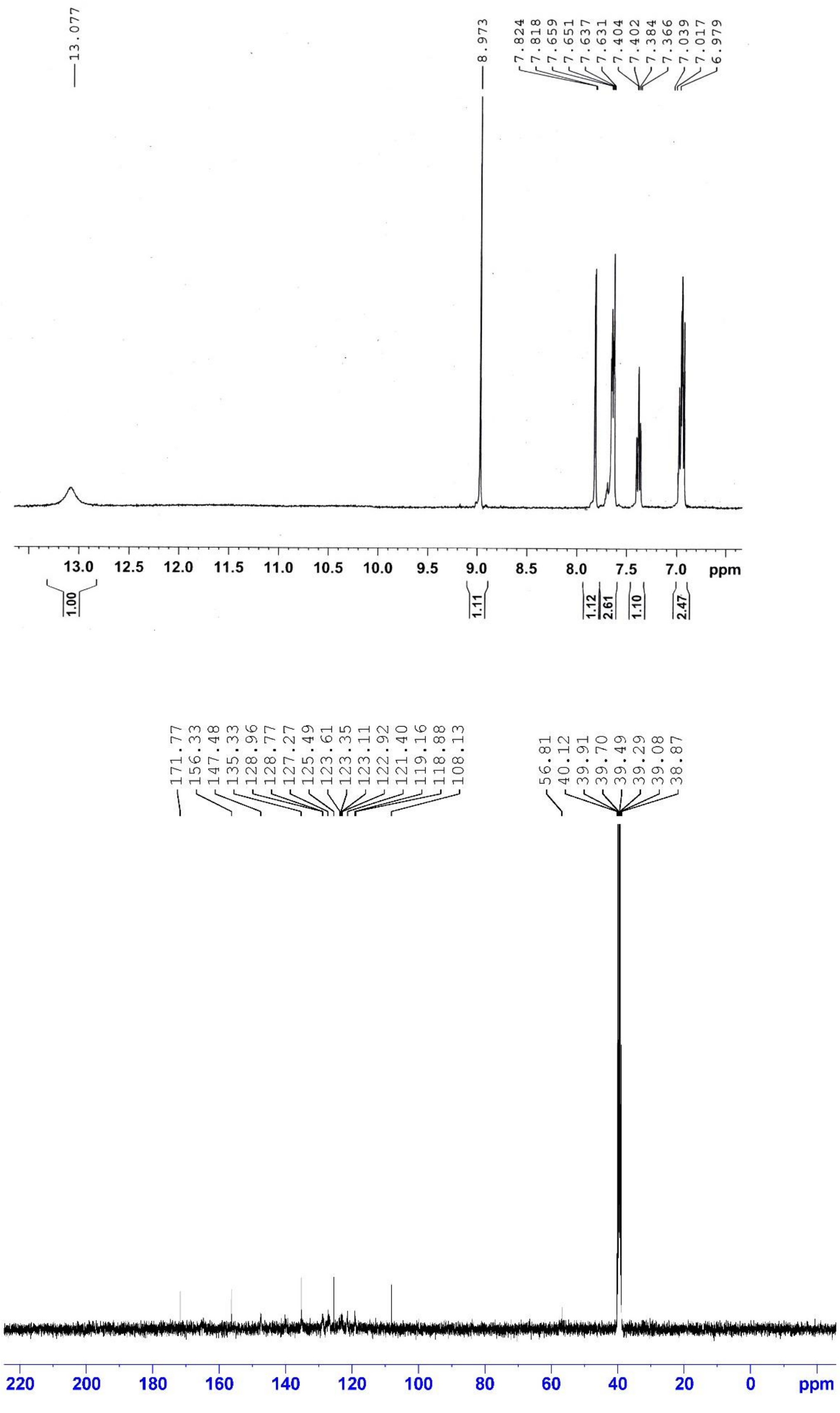

Fig. S2- ^{1}H and ^{13}C NMR spectra of complex **1** recorded in DMSO-d_6.

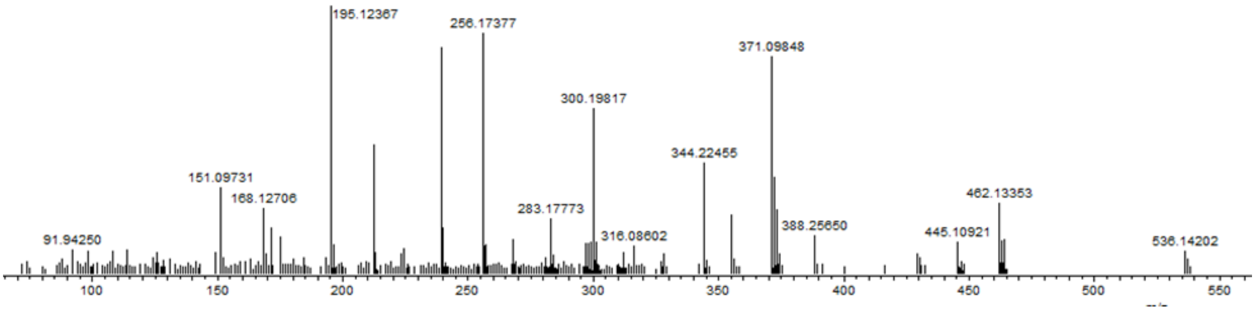

Fig. S3- Mass spectrum of complex **1.**

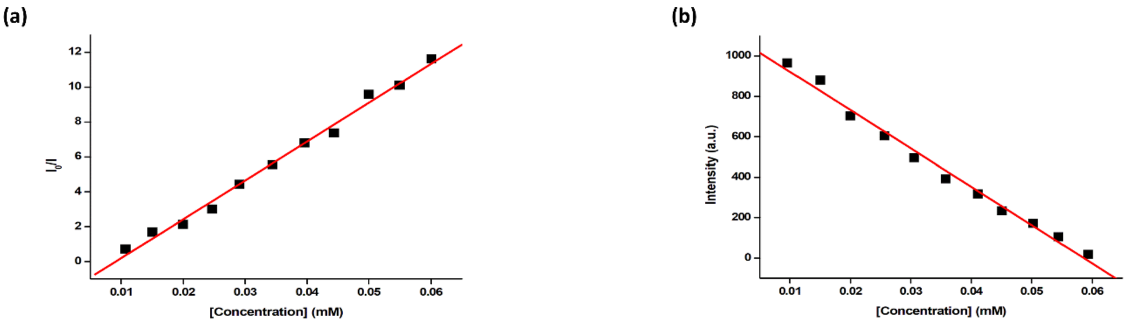

Fig S4- (a) Stern–Volmer plot for the fluorescence intensities of complex **1** upon addition of acetone, (b) The fitting curve of the luminescence intensity of complex **1** at different acetone concentrations.

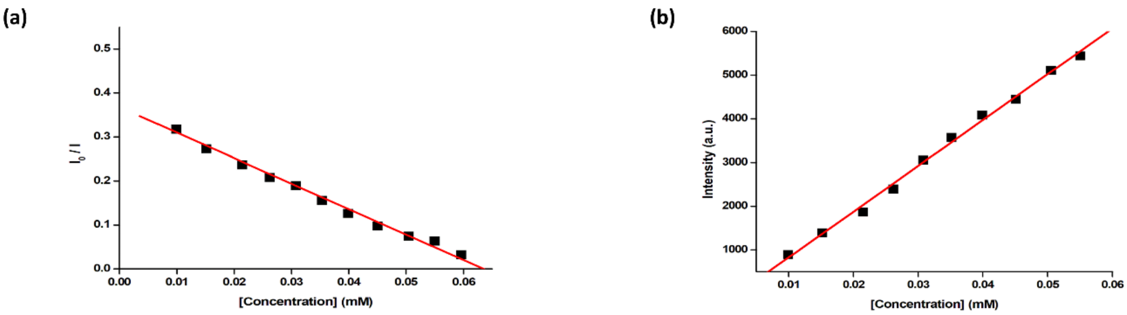

Fig S5- (a) Stern–Volmer plot for the fluorescence intensities of complex **1** upon addition of Ag^+ ions ((5×10^{-6} to 6.0×10^{-5} M), (b) The fitting curve of the luminescence intensity of complex **1** at different Ag^+ concentrations.

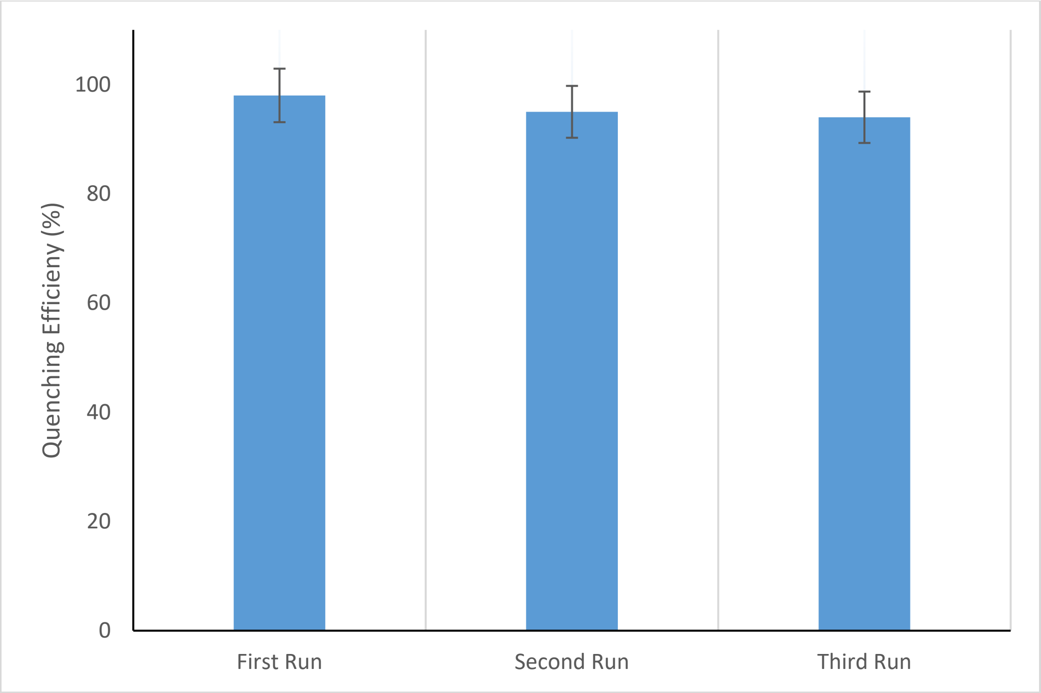

Fig S6- (a)

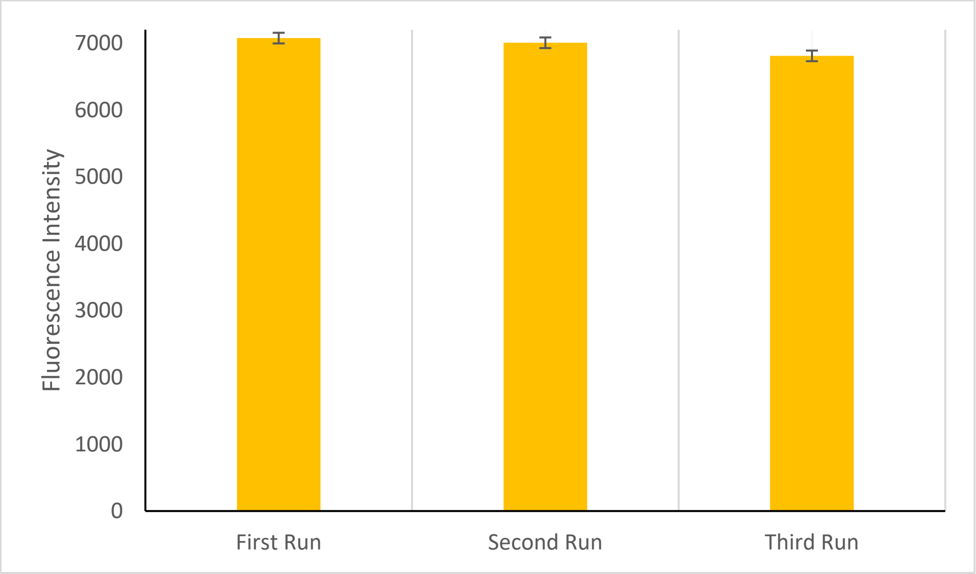

Fig S6- (b)

Fig S6- (a) Recycling test on sensing acetone in acetonitrile for complex **1.** (b) Recycling test on sensing Ag^{+} in acetonitrile for complex **1.**

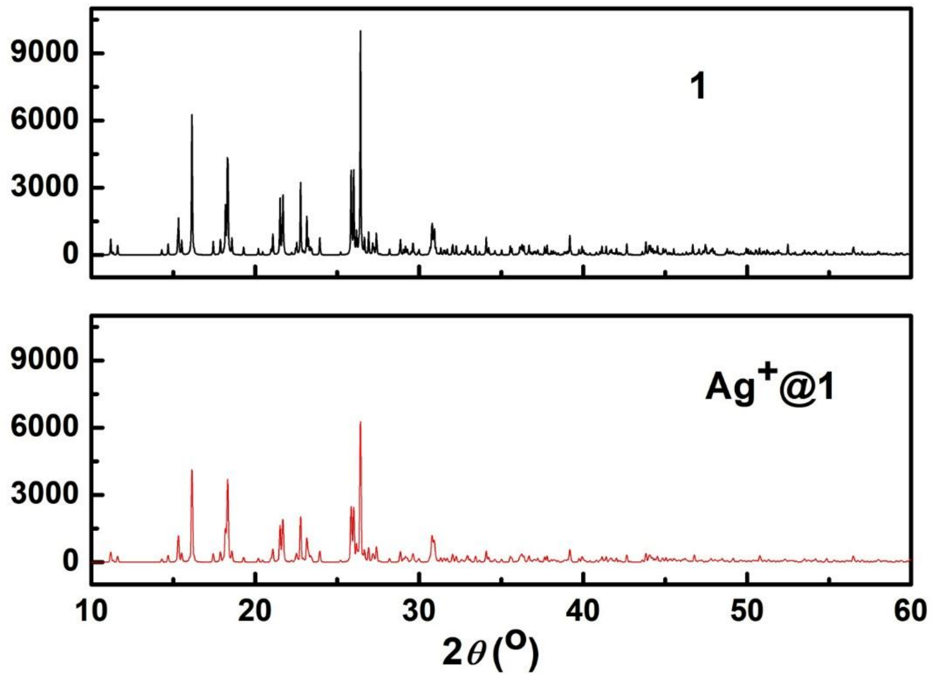

Fig. S7 The simulated Powder XRD patterns obtained from the single-crystal data of complex **1** and immersed with aqueous solution of Ag^+ at room temperature.
